# Interpreting biochemical control response rates with first-generation somatostatin analogues in acromegaly

**DOI:** 10.1007/s11102-015-0684-z

**Published:** 2015-10-30

**Authors:** Annamaria Colao, Renata S. Auriemma, Rosario Pivonello, Leandro Kasuki, Mônica R. Gadelha

**Affiliations:** Dipartimento di Medicina Clinica e Chirurgia, Università Federico II di Napoli, Via S Pansini 5, 80131 Naples, Italy; Endocrine Unit, Hospital Universitário Clementino Fraga Filho, Universidade Federal do Rio de Janeiro, Rio de Janeiro, Brazil

**Keywords:** Acromegaly, Octreotide, Lanreotide, Pasireotide, Somatostatin analogue, Response rate

## Abstract

**Context:**

The somatostatin analogues octreotide LAR and lanreotide Autogel have been evaluated for the treatment of acromegaly in numerous clinical trials, with considerable heterogeneity in reported biochemical response rates. This review examines and attempts to account for these differences in response rates reported in the literature.

**Evidence acquisition:**

PubMed was searched for English-language studies of a minimum duration of 24 weeks that evaluated ≥10 patients with acromegaly treated with octreotide LAR or lanreotide Autogel from 1990 to March 2015 and reported GH and/or IGF-1 data as the primary objective of the study.

**Evidence synthesis:**

Of the 190 clinical trials found, 18 octreotide LAR and 15 lanreotide Autogel studies fulfilled the criteria for analysis. It is evident from the protocols of these studies that multiple factors are capable of impacting on reported response rates. Prospective studies reporting an intention-to-treat analysis that evaluated medically naïve patients and used the composite endpoint of both GH and IGF-1 control were associated with lower response rates. The use of non-composite biochemical control endpoints, heterogeneous patient populations, analyses that exclude treatment non-responders, assay variability and prior responsiveness to medical therapy are just a few of the factors identified that likely contribute to higher success rates.

**Conclusions:**

The wide range of reported response rates with somatostatin analogues may be confusing and could lead to misinterpretation by both the patient and the physician in certain situations. Understanding the factors that potentially drive the variation in response rates should allow clinicians to better gauge treatment expectations in specific patients.

## Introduction

Acromegaly is almost always caused by a growth hormone (GH)-secreting pituitary tumor and is associated with increased morbidity and mortality [1]. Prompt treatment is essential in order to abrogate potentially life-threatening complications. Clinical practice guidelines advocate a multi-component therapeutic approach which includes lowering of both serum GH and insulin-like growth factor 1 (IGF-1) levels, tumor volume reduction, and amelioration of signs, symptoms and co-morbidities [1–3]. Somatostatin analogues, such as long-acting formulations of octreotide (octreotide LAR) and lanreotide (lanreotide Autogel), are recommended as first-line medical treatment for patients with acromegaly [2]. Octreotide LAR and lanreotide Autogel have been evaluated for the treatment of acromegaly in numerous clinical trials, with heterogeneity in reported biochemical response rates [4]. Studies of octreotide LAR prior to 2006 suggested that 50–70 % of patients with acromegaly respond to octreotide LAR [5–9]. In line with this, in a meta-analysis by Freda et al. [10] examining trials published before 2004, overall response rates for octreotide LAR were determined to be 57 % in terms of GH control and 67 % in terms of normalization of IGF-1. Additional studies of octreotide LAR [11, 12] and lanreotide Autogel [13, 14] published shortly after reported biochemical response rates as high as 70–80 %. More recently, however, response rates in prospective clinical trials of octreotide LAR, lanreotide Autogel and the multireceptor-targeted somatostatin analogue pasireotide have been substantially lower (17–41 %) [15–23].

The purpose of this review is to examine and attempt to account for the differences in response rates reported in the literature for first-generation somatostatin analogues. Where appropriate, examples will be provided to illustrate how various factors may affect reported outcomes. Clinical study results will also be discussed in order to help practitioners treating patients with acromegaly put results from published clinical trials into context.

## Selection of studies for review

PubMed was searched for English-language studies evaluating patients with acromegaly treated with octreotide LAR or lanreotide Autogel from 1990 (i.e., prior to the development of either drug formulation) to March 2015. Search terms used were “acromegaly and octreotide” and “acromegaly and lanreotide”; results were filtered for “clinical trial”. Studies that were included had ≥10 patients, a minimum duration of 24 weeks, used octreotide LAR or lanreotide Autogel as either first-line therapy or after previous surgery, radiotherapy or medical therapy, and reported GH and/or IGF-1 data as the primary objective of the study.

Of the 190 “clinical trials” found using “acromegaly and octreotide”, 18 studies fulfilled our criteria for analysis (Table 1) [5–9, 11, 12, 14–19, 24–28]. Of the 83 “clinical trials” found using “acromegaly and lanreotide”, 15 studies fulfilled our criteria for analysis (Table 2) [13, 14, 20–23, 29–36].

**Table 1:** Summary of octreotide LAR studies in acromegaly: study parameters and biochemical endpoints

Publication	n	Trial design	Trial duration	Patient population		Dose titration	GH and IGF-1 entry criteria	Target threshold values for GH and IGF-1	Biochemical control rates		
				Previous treatment	Prior response to SSA				GH n (%)	IGF-1 n (%)	GH + IGF-1 n (%)
Lancranjan et al. [8]	101	OL, P	30 mo	TSS + oct sc (n = 101)	83 pts were considered good responders to oct sc	Dose-ranging study: suppression of GH and IGF-1 assessed Biochemical control not defined			55 (55)^a^	66 (65)^b^	–
Davies et al. [7]	13	OL, P	1–3 yr	TSS (n = 2); RT (n = 1); TSS + RT (n = 4); Y implant (n = 1); de novo (n = 5)	Pts not selected for prior response	Dose-ranging study: suppression of GH and IGF-1 assessed Biochemical control not defined			6 (46)^c^	7 (54)^d^	–
Lancranjan et al. [9]	151	OL, P	12 mo	Oct sc (n = 49); TSS + oct sc (n = 56); RT + oct sc (n = 8); TSS + RT + oct sc (n = 38)	Only pts with GH < 10 µg/L during ≥4 wk treatment with oct sc were selected	Individualized according to the mean GH after the second dose	GH > 2 µg/L after OGTT and elevated IGF-1	GH ≤ 2.5 µg/L IGF-1 normal	104 (69)	98 (65)	–
Colao et al. [5]	36	OL, P	24 mo	TSS (n = 5); TSS + oct sc (n = 5); TSS + oct LAR (n = 3); TSS + lan SR (n = 8); de novo (n = 15)	16/21 pts with previous surgery received oct sc	Uptitrated in 15 pts with GH > 5 µg/L	GH > 2 µg/L after OGTT and elevated IGF-1 for age	GH ≤ 2.5 µg/L IGF-1 normal	20 (56)	19 (53)	–
Cozzi et al. [6]	110	OL, Ret	18–54 mo	TSS + oct LAR (n = 29); RT + oct LAR (n = 7); TSS + RT + oct LAR (n = 23); oct sc (n = 39); de novo (n = 12)	Pts had to have a GH and/or IGF-1 decrease of at least 20 % after a 6-month trial with oct LAR	Individualized to achieve normal age-adjusted IGF-1 levels and GH <2.5 µg/L	GH > 1 µg/L after OGTT and elevated IGF-1 for age	GH < 2.5 µg/L IGF-1 normal	79 (72)^e^	83 (75)^e^	–
Ayuk et al. [24]	91^f^	OL, Ret	48 wk	Oct sc (n = 34); TSS + oct sc (n = 29); RT + oct sc (n = 5); TSS + RT + oct sc (n = 23)	Only patients who completed 48 wk treatment were considered	Individualized according to the mean GH after the second dose	GH > 2 µg/L after OGTT and elevated IGF-1	GH < 2.0 µg/L	61 (67)	48 (72)	–
Jallad et al. [25]	80	OL, P	12 mo	TSS + oct LAR (n = 24); TSS + RT + oct LAR (n = 28); lan (n = 14); DA (n = 1); de novo (n = 13)	Pts not selected for prior response	Individualized to achieve normal age-adjusted IGF-1 levels	GH > 1 µg/L after OGTT and elevated IGF-1 for age and sex	GH < 2.5 µg/L IGF-1 normal	34 (43)	20 (25)	–
Cozzi et al. [12]	67	OL, P	6–108 mo	De novo (n = 67)	Pts not selected for prior response	Individualized at 3- to 6-mo intervals if GH or IGF-1 levels remained elevated	GH > 1 µg/L after OGTT and elevated IGF-1 for age	GH < 2.5 µg/L IGF-1 normal	46 (69)^e^	47 (70)^e^	38 (57)^e^
Colao et al. [11]	56	OL, Ret	24 mo	De novo (n = 56)	16 pts were excluded from the efficacy analysis because of lack of response before 24 mo	Uptitration in pts with IGF-1 levels above the normal range and/or GH > 2.5 mg/L	GH > 1 µg/L after OGTT and elevated IGF-1 for age	GH ≤ 2.5 µg/L IGF-1 normal	48 (86)	47 (84)	45 (80)
Mercado et al. [18]	98	OL, P	48 wk	De novo (n = 98)	Pts not selected for prior response	Uptitration in pts with GH > 2.5 μg/L or IGF-1 above the adjusted normal range at 4 and 6 mo	GH > 1 µg/L after OGTT and IGF-1 > 97th percentile of the normal range for age and sex	GH ≤ 2.5 µg/L IGF-1 normal	30 (31)	23 (23)	17 (17)
Andries et al. [28]	12	OL, R, P, CO	12 mo	TSS alone (n = 4); TSS + radiotherapy (n = 3); oct LAR (n = 11)	4 pts had control of both GH and IGF-1 at study entry	–	–	GH < 0.38 µg/L IGF-1 normal	5 (42)	5 (42)	4 (33)
Colao et al. [16]	48	OL, R, P	48 wk	De novo (n = 48)	Pts not selected for prior response	Uptitration in pts with mean GH >2.5 μg/L and/or elevated IGF-1 at 3 and 6 mo	GH > 1 μg/L after OGTT and IGF-1 > 97th percentile for age and sex	GH ≤ 2.5 µg/L IGF-1 normal	–	–	13 (27)
Ghigo et al. [26]	56	OL, R, P	12 mo	All pts naïve to medical therapy and RT; TSS in an unreported number of pts	Pts not selected for prior response	Uptitration in pts with IGF-1 within 20 % below ULN or > ULN every 16 wk	GH > 1 μg/L after OGTT and IGF-1 ≥ 1.3xULN	IGF-1 normal	–	19 (34)	–
Oki et al. [27]	30	OL, Ret	24 mo	TSS alone (n = 12); TSS + DA (n = 11); TSS + radiotherapy (n = 1); TSS + radiotherapy + DA (n = 5); de novo (n = 1)	Pts not selected for prior response	Uptitration in pts with mean GH >2.5 μg/L and/or elevated IGF-1 every 3 mo	–	GH ≤ 2.5 µg/L IGF-1 normal	17 (57)	16 (53)	11 (37)
Karaca et al. [17]	11	R, P	12 mo	De novo (n = 11)	Pts not selected for prior response	Dose titration according to biochemical response at 3, 6 and 12 mo	–	GH ≤ 2.5 µg/L IGF-1 normal	9 (82)	3 (27)	3 (27)
Carlsen et al. [15]	32	R, P	24 wk	De novo (n = 11)	Pts not selected for prior response	Fixed dose	GH > 5 mIU/L after OGTT	GH ≤ 2 mIU/L during OGTT IGF-1 ≤ ULN	11 (34)	10 (31)	7 (22)
Tutuncu et al. [14]	36	OL, Ret	18 mo	TSS (n = 36)	Pts not selected for prior response	Dose titration according to biochemical response at 6 mo	GH > 1 μg/L after OGTT or mean GH > 5 µg/L and IGF-1 > ULN for age and sex	GH ≤ 2.5 µg/L IGF-1 normal	24 (67)	24 (67)	23 (64)
Colao et al. [19]	182	R, P, DB	12 mo	TSS (n = 80); RT (n = 1); de novo (n = 101)	Pts not selected for prior response	Optional uptitration in pts with mean GH ≥2.5 μg/L and/or IGF-1 > ULN at 3 and 7 mo	GH > 1 μg/L after OGTT or mean GH > 5 µg/L and IGF-1 > ULN for age and sex	GH < 2.5 µg/L IGF-1 normal	94 (52)	43 (24)	35 (19)

*CO* crossover, *DA* dopamine agonist, *DB* double blind, *GH* growth hormone, *IGF-1* insulin-like growth factor 1, *lan* lanreotide, *LAR* long-acting release, *mo* month, *oct* octreotide, *OGTT* oral glucose tolerance test, *OL* open label, *P* prospective, *pts* patients, *R* randomized, *Ret* retrospective, *RT* radiotherapy, *sc* subcutaneous, *SR* slow release, *SSA* somatostatin analogues, *TSS* transsphenoidal surgery, *ULN* upper limit of normal, *wk* week, *yr* year, *Y* yttrium

^a^GH < 2 µg/L

^b^IGF-1 to within normal range

^c^GH < 5 mU/L

^d^IGF-1 < 65 nmol/L

^e^Response at last follow-up

^f^Only patients who completed 48 weeks of treatment in the Lancranjan et al. study [9] and had diagnostic GH (n = 91) or IGF-1 (n = 67) data were considered for this retrospective analysis

**Table 2:** Summary of lanreotide Autogel studies in acromegaly: study parameters and biochemical endpoints

Publication	n	Trial design	Trial duration	Patient population		Dose titration	GH and IGF-1 entry criteria	Target threshold values for GH and IGF-1	Biochemical control rates		
				Previous treatment	Prior response to SSA				GH n (%)	IGF-1 n (%)	GH + IGF-1 n (%)
Caron et al. [29]	130	OL, P	12 mo	TSS (n = 99); RT (n = 57); lan SR (n = 130); lan ATG (n = 130)	All pts had completed a 3-month study of lan ATG in which pts were selected based on response to lan SR	Dose titration according to biochemical response at study entry, 4 and 8 mo	–	GH < 2.5 µg/L IGF-1 normal	83 (64)	62 (48)	52 (40)
Alexopoulou et al. [30]	25	OL, P	24 wk	TSS (n = 13); RT (n = 5); oct LAR (n = 25)	All pts had been treated with oct LAR at a fixed dose for ≥6 mo	Dose titration according to biochemical response at 8 wk	–	GH < 2.5 µg/L IGF-1 normal	12 (48)	13 (52)	–
Lucas et al. [36]	98	OL, P	12–30 wk	TSS (n = 76); radiotherapy (n = 53); lan SR (n = 98)	All pts had been treated with lan SR for ≥ 2 mo immediately prior to study entry	Fixed dose	GH > 2 µg/L after OGTT and IGF-1 > ULN for age and sex	GH < 2.5 µg/L IGF-1 normal	53 (54)	55 (56)	39 (40)
Ronchi et al. [31]	23	OL, P	34–42 wk	TSS (n = 16); oct LAR (n = 23)	Only pts with GH reduction of >50 % during 6–18 mo treatment with oct LAR were selected	Dose titration according to biochemical response at 18 wk	GH reduction of >50 % during 6–18 mo treatment with oct LAR	GH < 2.5 µg/L IGF-1 normal	13 (57)	9 (39)	7 (30)
Chanson et al. [13]	63	OL, P	48 wk	TSS (n = 37); RT (n = 12); prior medical therapy (n = 49)	Pts not selected for prior response	Dose titration according to biochemical response at 16 and 32 wk	IGF-1 ≥ 1.3xULN	GH < 2.5 µg/L IGF-1 normal	53 (84)^a^	27 (43)^a^	24 (38)
Attanasio et al. [32]	27	OL, P	12 mo	TSS (n = 8); de novo (n = 19)	Pts not selected for prior response	Individualized every mo according to biochemical response	GH > 1 μg/L after OGTT and IGF-1 > ULN for age and sex	GH < 2.5 µg/L IGF-1 normal	11 (41)	14 (52)	10 (37)
Andries et al. [28]	12	OL, R, P, CO	12 mo	TSS alone (n = 4); TSS + radiotherapy (n = 3); oct LAR (n = 11)	4 pts had control of both GH and IGF-1 at study entry	–	–	GH < 0.38 µg/L IGF-1 normal	5 (42)	6 (50)	5 (42)
Colao et al. [33]	26	OL, P	12 mo	De novo (n = 26)	Pts not selected for prior response	Dose titration according to biochemical response at 12 wk	GH > 1 μg/L after OGTT or mean GH > 2.5 µg/L and IGF-1 > ULN for age and sex	GH ≤ 5 mU/L IGF-1 normal	15 (58)	15 (58)	14 (54)
Lombardi et al. [34]	63	OL, P	48–52 wk	TSS (n = 12); de novo (n = 39)^b^	Pts not selected for prior response	Dose titration according to biochemical response at 24 wk	GH > 1 μg/L after OGTT or mean GH > 5 µg/L and IGF-1 > ULN for age and sex	GH < 2.5 µg/L IGF-1 normal	32 (51)	19 (30)	18 (29)
Melmed et al. [20]	107	OL, R, P	52 wk	TSS (n = 59); RT (n = 12); lan SR (n = 21); oct LAR (n = 27); oct sc (n = 4); DA (n = 4); de novo (n = 15)	Pts not selected for prior response	Dose titration according to biochemical response at 16 and 32 wk	GH > 3–5 µg/L	GH ≤ 2.5 µg/L IGF-1 normal	55 (51)	62 (58)^c^	44 (41)^c^
Salvatori et al. [21]	26	OL, P	6 mo	TSS (n = 22); oct LAR (n = 4); oct sc (n = 1); DA (n = 6); no prior medical therapy (n = 15)	Pts not selected for prior response	Dose titration according to biochemical response at 16 wk	–	GH < 1.0 µg/L post-OGTT IGF-1 normal	9 (35)	10 (38)^d^	7 (27)^d^
Schopohl et al. [35]	37	OL, P	24–48 wk	Oct LAR (n = 37); TSS in an unreported number of pts	Pts were adequately treated with oct LAR for ≥6 mo (IGF-1 ≤ 1.3xULN)	Dose titration after 3 injections to lan ATG every 28, 42 or 56 days	IGF-1 ≤ 1.3xULN while on oct LAR	GH < 2.0 µg/L IGF-1 normal	25 (68)^e^	22 (59)	–
Tutuncu et al. [14]	32	OL, Ret	18 mo	TSS (n = 32)	Pts not selected for prior response	Dose titration according to biochemical response at 6 mo	GH > 1 μg/L after OGTT or mean GH > 5 µg/L and IGF-1 > ULN for age and sex	GH < 2.5 µg/L or < 1 µg/L post-OGTT IGF-1 normal	25 (78)	25 (78)	25 (78)
Annamalai et al. [22]	30	OL, P	24 wk	De novo (n = 30)	Pts not selected for prior response	Uptitration in pts with mean GH ≥ 2.5 μg/L and/or IGF-1 > ULN at 3 mo	GH > 0.4 μg/L after OGTT and IGF-1 > ULN for age and sex	GH < 1.0 µg/L IGF-1 ≤ 1.1xULN	12 (40)	12 (40)	10 (33)
Shimatsu et al. [23]	32	OL, R, P	24 wk	TSS (n = 26), RT (n = 4), medical therapy (n = 13)	Pts not selected for prior response	Fixed dose	GH > 1.7–2.8 µg/L	GH < 2.5 µg/L IGF-1 normal	17 (53)	14 (44)	13 (41)
Shimatsu et al. [23]	32	OL, P	12 mo	TSS (n = 27), RT (n = 6), medical therapy (n = 20)	8 patients had completed a previous study of lan ATG	Uptitration in pts with mean GH ≥1.0 μg/L and/or IGF-1 > ULN at 4 and 8 mo	GH > 2.5 µg/L	GH < 2.5 µg/L IGF-1 normal	15 (47)	17 (53)	13 (41)

*ATG* Autogel, *CO* crossover, *DA* dopamine agonist, *GH* growth hormone, *IGF-1* insulin-like growth factor 1, *lan* lanreotide, *LAR* long-acting release, *mo* month, *oct* octreotide, *OGTT* oral glucose tolerance test, *OL* open label, *P* prospective, *pts* patients, *R* randomized, *Ret* retrospective, *RT* radiotherapy, *sc* subcutaneous, *SR* slow release, *SSA* somatostatin analogues, *TSS* transsphenoidal surgery, *ULN* upper limit of normal, *wk* week, *yr* year

^a^35 % of patients had GH ≤ 2.5 µg/L at baseline

^b^An unreported number of patients had received ≤3 months’ presurgical treatment with SSA

^c^IGF-1 levels were normal in 10 % of patients at baseline

^d^IGF-1 levels were normal in 15 % of patients at baseline

^e^At baseline, 28 patients (76 %) had GH levels < 2.0 µg/L

## Factors that may impact on response rates

A recent meta-analysis by Carmichael et al. [37] that looked into how various aspects of acromegaly clinical trial methodology impact on reported response rates to somatostatin analogues concluded that year of publication, study duration and prior somatostatin analogue use significantly affected response rates. This analysis looked at how single factors might affect outcomes, rather than a combination of factors. There are many aspects to clinical studies that can potentially affect reported response rates, including the definition of response, the characteristics of the enrolled patients, whether or not all of the patients recruited into the study were included in the analysis, and whether the study was prospective or retrospective, among others. Most studies will be associated with a number of these factors, all of which can affect reported response rates.

### Patient population

Clinical trials of any drug in any disease area select patients for enrollment into the study through a series of inclusion and exclusion criteria. These criteria are necessary to ensure that the correct patient population is treated for the hypothesis being examined, and in acromegaly may include such factors as sex, age, previous treatment history (e.g., transsphenoidal surgery, radiotherapy or medical therapy), and the presence or absence of other medical conditions.

Evaluating a drug effect in different subpopulations of patients with acromegaly may result in differing outcomes. In early studies of octreotide LAR and lanreotide Autogel, most studies included patients who were known responders to the drug (Tables 1, 2). As octreotide LAR and lanreotide Autogel were new formulations of the available drugs, this inclusion criterion was chosen to show that the new formulations were as effective as the older formulations in order to gain market approval. However, if taken out of context, the reported response rates may appear relatively high when compared with more recent results in which patients were not selected for prior responsiveness. For example, early studies of octreotide LAR (Lancranjan et al. [8], [9]) and lanreotide Autogel (Caron et al. [29]), which included patients known to be responsive to subcutaneous (sc) octreotide immediate release and lanreotide slow release (SR), respectively, showed GH response rates of 55–69 % [8, 9, 29] and IGF-1 response rates of 48–65 % [8, 9, 29]. Most of the patients in these studies had also received prior transsphenoidal surgery and/or radiotherapy.

More recently, somatostatin analogues have been evaluated as first-line therapy, in which the enrolled patients had not previously received any treatment for acromegaly. The percentage of patients achieving the composite endpoint of both GH and IGF-1 control after 1 year of treatment in prospective studies of patients with previously untreated acromegaly was 17–27 % with octreotide LAR [16–18] and 33–54 % with lanreotide Autogel [22, 33]. Additionally, the recent randomized, double-blind study of pasireotide LAR versus octreotide LAR by Colao et al. [19] in patients who were either de novo or had received transsphenoidal surgery (but no medical therapy and no radiotherapy within 10 years) reported that 19 % of octreotide LAR recipients and 31 % of pasireotide LAR recipients achieved both GH and IGF-1 control after 1 year of treatment. When patients were stratified by prior treatment, the response rates in de novo patients were 17 and 26 % for octreotide LAR and pasireotide LAR, respectively, compared with 22 and 39 % in patients who had received prior surgery. Interestingly, in a 52-week study of lanreotide Autogel, 55 % of patients who had received prior treatment with a somatostatin analogue achieved biochemical control, compared with 20 % of somatostatin-analogue-naïve patients [20].

Although there are other factors that may have contributed to the relatively low response rates in these more recent studies, these results suggest that response rates in studies of patients with de novo acromegaly are generally lower than response rates in prospective studies of patients who have previously received transsphenoidal surgery and/or radiotherapy and/or somatostatin analogues. This is consistent with a number of studies that showed that by reducing tumor mass and, therefore, decreasing basal GH secretion, subsequent control of GH and IGF-1 levels with somatostatin analogues may be improved [38–42].

### Heterogeneity in the definition of biochemical control

**Fig. 1:**
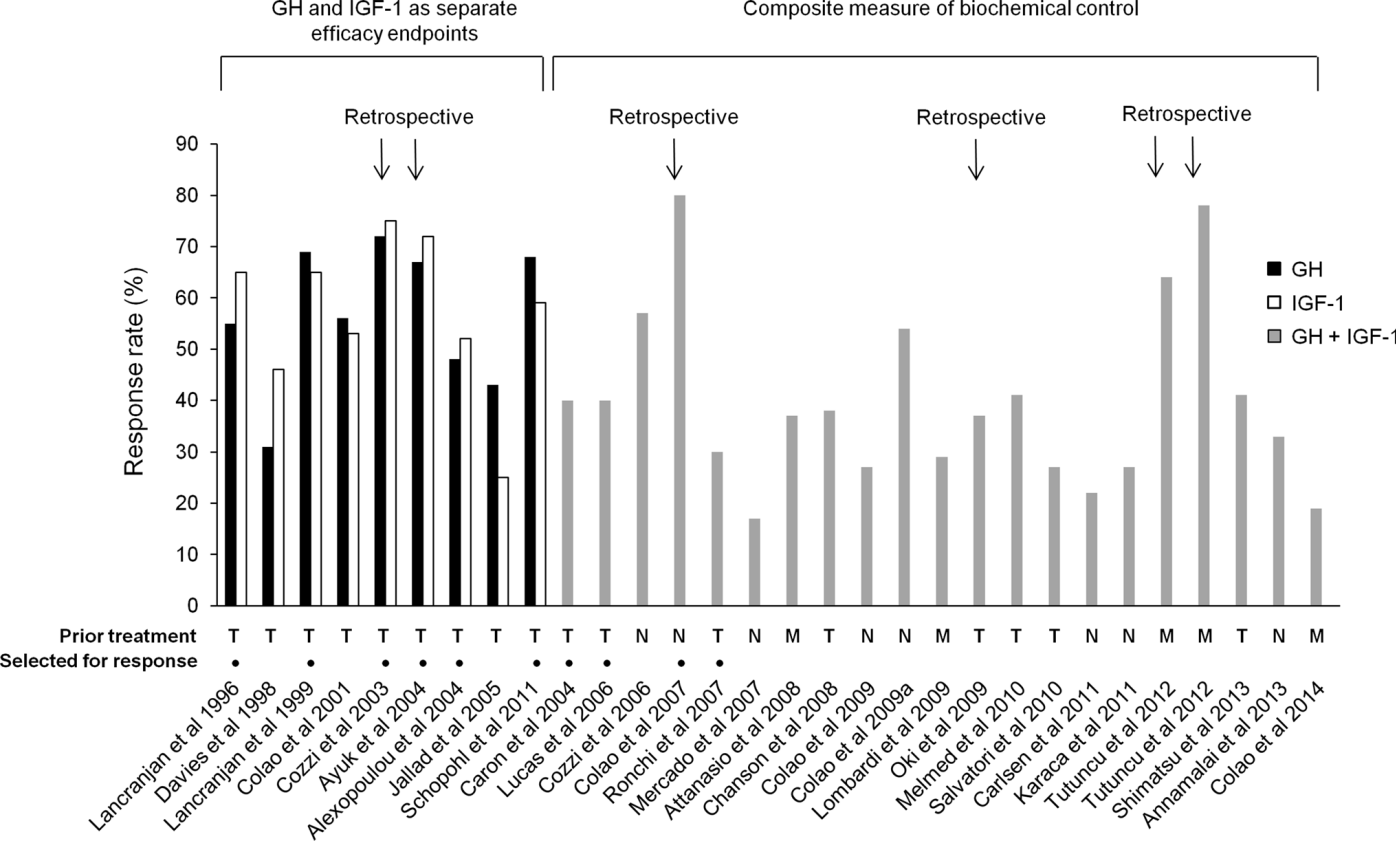
Biochemical response rates to octreotide LAR and lanreotide Autogel reported in the medical literature by publication year and stratified according to whether GH and IGF-1 were reported as separate efficacy endpoints or as a composite efficacy endpoint. M = prior surgery but medically naive; N = treatment naive; T = previously treated with surgery and/or radiotherapy and/or medical therapy

The two parameters of biochemical control, suppression of excess GH secretion to predefined threshold levels and normalization of serum IGF-1 levels, are used as the primary markers of efficacy in most clinical trials of somatostatin analogues in patients with acromegaly. From the studies fulfilling the search criteria (Tables 1, 2), target threshold levels are generally similar across all the studies (the majority use GH ≤ 2.5 µg/L and normal IGF-1 for age and sex). However, studies published before 2006 generally reported response rates for GH and IGF-1 separately [5–9, 24, 25, 30], rather than the percentage of patients who achieved control of both GH and IGF-1 levels (Fig. 1).

Evaluation of GH or IGF-1 separately, rather than as a composite endpoint, was employed in ‘early’ clinical studies (1996–2006) [5–9, 24, 25, 30] and resulted in response rates that were relatively high compared with those in studies reported after 2006 (Fig. 1). The literature around this ‘early’ period began to report that patients with GH levels ≤2.5 µg/L did not necessarily achieve normalization of IGF-1 levels [43]. Indeed, approximately 25 % [6, 43] of patients were suggested to have discrepant GH and IGF-1 control (control of GH without IGF-1 control, or vice versa). As a consequence, the measurement of a composite efficacy endpoint of control of both GH and IGF-1 levels was advocated [44] and subsequently recommended by clinical practice guidelines for assessing biochemical response [1]. These findings likely prompted a shift away from only reporting GH and IGF-1 separately towards the more stringent composite measure and, as might be expected, the majority of the more recent studies (2007–2014) [6, 11, 12, 15–19] are generally associated with relatively low response rates (Fig. 1). Thus, heterogeneity in the definition of biochemical control may be a contributing factor giving rise to differences in reported response rates.

### Study design and nuances of the study protocol

#### Prospective versus retrospective

Although both prospective and retrospective studies provide valuable data, retrospective studies are more likely to contain elements of bias and confounding. One of the main elements of bias associated with retrospective studies is the inclusion of only those patients who completed a certain duration of treatment and were not lost to follow-up or did not discontinue treatment because of adverse events or other reasons. For example, the retrospective study by Cozzi et al. [6], which reported a GH response rate of 72 % and an IGF-1 response rate of 75 %, excluded an unreported number of patients who received octreotide LAR for less than 18 months. Had all patients who started on octreotide LAR been included in the analysis, the reported response rates may have been lower, as patients not reaching 18 months of therapy may have discontinued treatment because of non-response. Similarly, the retrospective comparison by Tutuncu et al. [14] of octreotide LAR and lanreotide Autogel in patients who had failed surgery reported that 64 % of octreotide LAR patients and 78 % of lanreotide Autogel patients had biochemical control after 18 months of treatment. However, the analysis retrospectively excluded all patients who had required a second operation or who had received additional medical treatment within 18 months of the initial surgery (i.e., probable non-responders to octreotide or lanreotide), thus artificially increasing response rates. As can be seen in Fig. 1, retrospective studies generally result in higher response rates, and the main reason is usually the retrospective exclusion of non-responders from the analysis.

#### Per protocol or intention to treat

However, many prospective studies also exclude patients who did not complete the study. For example, the prospective, 1-year follow-up study of lanreotide Autogel by Caron et al. [29] enrolled 130 patients previously treated with surgery and/or lanreotide Autogel, yet nine patients were excluded from the final analysis of biochemical control. Although the reasons for exclusion from the analysis may appear valid from a clinical practice perspective, for example, patients dropped out before the first efficacy analysis or withdrew consent to remain on treatment, these patients should be included as non-responders in an intention-to-treat (ITT) analysis. Therefore, although the study reported that 43 % (52/121) of the ITT population achieved biochemical control [29], the actual response rate was 40 % (52/130) (Table 2).

An efficacy analysis conducted on either the per-protocol (PP) or ITT population can produce significantly different outcomes. In an ITT analysis, all patients who were enrolled are considered part of the study, whether they receive treatment, complete the study or not. A PP analysis, however, is based on the population of patients who completed the clinical trial without any major protocol violations. For example, the study by Mercado et al. [18] reported results based on the PP population. This prospective, multicenter study evaluated octreotide LAR as first-line therapy in 98 previously untreated patients for 1 year. At month 12, 30 patients were excluded from the efficacy analysis because of either major protocol violations or early discontinuation; therefore, 68 patients formed the PP population. Seventeen patients achieved biochemical control (GH ≤ 2.5 μg/L and normalization of IGF-1 levels), which corresponds to a response rate of 25 % (17/68) based on the PP analysis, but 17.3 % (17/98) based on analysis of the ITT population (Table 1). Thus, analysis of the PP population usually leads to the reporting of higher response rates than an analysis of all patients who were intended to be treated.

#### Definition of responders and non-responders

Open-ended studies that report patients’ response at last follow-up generally result in a higher overall response rate than studies with a fixed time point to evaluate response. Although the definition of response may be the same with respect to the biochemical parameters (e.g., GH ≤ 2.5 µg/L and normal IGF-1), the time point at which biochemical control is measured may differ. In the study by Cozzi et al. [12], 56.7 % of previously untreated patients were reported to have achieved biochemical control (GH ≤ 2.5 µg/L and normal IGF-1). Patients in this study were followed up for 6–108 months, and their response at last follow-up was considered. As such, the 56.7 % of patients who were considered responders had achieved a response at some point between months 6 and 108 and so should not be compared with studies of a similar patient population that reported response rates at a specific time point, for example, at month 12 of treatment.

Furthermore, the pre-specified definition of biochemical control should be adhered to during analysis of the results. Like most studies, the randomized, double-blind study of pasireotide LAR versus octreotide LAR by Colao et al. [19] defined biochemical control as GH < 2.5 µg/L and normal IGF-1 at month 12. In this study, a patient with IGF-1 *below* the lower limit of normal was not considered a responder for the primary analysis because IGF-1 was abnormal. The consequence of this protocol definition is best exemplified by a post hoc analysis which included these patients with IGF-1 below the lower limit of normal as responders: response rates for octreotide LAR and pasireotide LAR increased from 19.2 to 20.9 % and from 31.3 to 35.8 %, respectively [19]. These examples emphasize the importance of appreciating the definitions of responders in study protocols and how they are treated in the primary analysis.

### Assay variability

A wide range of immunoassays are used for the assessment of GH and IGF-1 levels. However, considerable heterogeneity in assay characteristics exists, which may lead to variability in results. This assay variability can be largely classified into two categories: cross-sectional variation, where different assays give differing results [45, 46]; and longitudinal variation, in which assays are unstable over time [47]. The lack of standardization between assays is of such concern that a 2011 consensus statement on the standardization and evaluation of GH and IGF-1 assays was developed as part of an international effort to harmonize GH and IGF-1 assays [48]. For the purposes of the current review, an analysis of the assays used in each study has not been undertaken. However, it is likely that some of the variability in GH and IGF-1 response rates seen in studies in this review is due to assay variability.

## Response rates in a clinical trial setting: summary

Using representative examples of different clinical trial protocols evaluating octreotide LAR and lanreotide Autogel as a treatment for acromegaly, it is evident that multiple factors are capable of impacting on reported response rates. Prospective studies reporting an ITT analysis that evaluated medically naïve patients, used the composite endpoint of both GH and IGF-1 control, and used a fixed time point to evaluate response were associated with lower response rates. The use of less stringent non-composite biochemical control endpoints, heterogeneous patient populations, study protocols that exclude treatment non-responders from the efficacy analysis, and prior responsiveness to medical therapy are just a few of the factors identified that likely contribute to higher success rates. This review does not attempt to place emphasis on any one particular factor over another, but it serves instead to raise awareness of the dangers of interpreting biochemical control response rates without first carefully considering how the study design, patient population and statistical analysis might impact on the data. Multivariate statistical analyses exploring the differences in response rates between studies would help to quantify the impact these factors may have on reported outcomes, and such an analysis is encouraged. With this in mind, it is perhaps unsurprising that response rates to somatostatin analogues, or to agents of any class of drug, in published clinical trials vary considerably.

## Response rates in clinical practice

In clinical practice, biochemical response rates to somatostatin analogues are likely to fall somewhere between the rates observed in the early clinical trials and the rates observed in more recent trials. Many patients seen in the clinic are post-surgical or have had successful prior treatment with a somatostatin analogue, rather than being treatment naïve. Prior to treatment with somatostatin analogues, patients may also be ‘pre-selected’ for potential responsiveness with an acute octreotide suppression test [49]. The clinical setting allows for dose adjustments at the discretion of the physician and long-term treatment, both of which provide opportunities for higher response rates, similar to long-term, open-label studies. The choice of parameters used to gauge clinical response also plays a significant role in perceived treatment success; both GH and IGF-1 levels together as a composite measure should be used to assess biochemical response [2, 50].

Finally, similar to the differences in clinical trials of reporting data from patients who discontinue treatment (e.g., ITT vs. PP analysis), clinicians (including those from specialist pituitary centers) sometimes might remember only those patients who have responded to treatment, rather than those who have transitioned to another treatment or care provider. As a result, clinicians should be cautious of comparing response rates in real-world clinical scenarios with response rates obtained from a controlled trial setting.

As the mechanisms involved in the resistance to somatostatin analogues become better understood, it should be possible to predict which patients will respond to different medical therapies based on biomarkers. Therefore, a more successful outcome may be observed if individualized treatment is based on information such as the somatostatin receptor subtype profile, aryl hydrocarbon receptor-interacting protein (AIP) expression, and T2 intensity on magnetic resonance imaging, among others [51, 52]. This would result in higher response rates, because those patients who would not have responded to treatment with a particular agent would not be unnecessarily treated.

## Concluding remarks

As acromegaly is a rare disorder, even experienced endocrinologists may seldom treat a patient with acromegaly and therefore rely on the medical literature to inform treatment decision making. However, the wide range of reported response rates with octreotide LAR and lanreotide Autogel may be confusing and could lead to misinterpretation by both the patient and the physician in certain situations. Understanding the factors that potentially drive the discordance in response rates, as reported in this review, should allow clinicians to better gauge treatment expectations in specific patients.
